# Logarithmic and Power Law Input-Output Relations in Sensory Systems with Fold-Change Detection

**DOI:** 10.1371/journal.pcbi.1003781

**Published:** 2014-08-14

**Authors:** Miri Adler, Avi Mayo, Uri Alon

**Affiliations:** Department of Molecular Cell Biology, Weizmann Institute of Science, Rehovot, Israel; ETH Zurich, Switzerland

## Abstract

Two central biophysical laws describe sensory responses to input signals. One is a logarithmic relationship between input and output, and the other is a power law relationship. These laws are sometimes called the Weber-Fechner law and the Stevens power law, respectively. The two laws are found in a wide variety of human sensory systems including hearing, vision, taste, and weight perception; they also occur in the responses of cells to stimuli. However the mechanistic origin of these laws is not fully understood. To address this, we consider a class of biological circuits exhibiting a property called fold-change detection (FCD). In these circuits the response dynamics depend only on the relative change in input signal and not its absolute level, a property which applies to many physiological and cellular sensory systems. We show analytically that by changing a single parameter in the FCD circuits, both logarithmic and power-law relationships emerge; these laws are modified versions of the Weber-Fechner and Stevens laws. The parameter that determines which law is found is the steepness (effective Hill coefficient) of the effect of the internal variable on the output. This finding applies to major circuit architectures found in biological systems, including the incoherent feed-forward loop and nonlinear integral feedback loops. Therefore, if one measures the response to different fold changes in input signal and observes a logarithmic or power law, the present theory can be used to rule out certain FCD mechanisms, and to predict their cooperativity parameter. We demonstrate this approach using data from eukaryotic chemotaxis signaling.

## Author Summary

One of the first measurements an experimentalist makes to understand a sensory system is to explore the relation between input signal and the systems response amplitude. Here, we show using mathematical models that this measurement can give important clues about the possible mechanism of sensing. We use models that incorporate the nearly-universal features of sensory systems, including hearing and vision, and the sensing pathways of individual cells. These nearly-universal features include exact adaptation-the ability to ignore prolonged input stimuli and return to basal activity, and fold-change detection- response to relative changes in input, not absolute changes. Together with information on the input-output relationship-e.g. is it a logarithmic or a power law relationship-we show that these conditions provide enough constraints to allow the researcher to reject certain circuit designs; it also predicts, if one assumes a given design, one of its key parameters. This study can thus help unify our understanding of sensory systems, and help pinpoint the possible biological circuits based on physiological measurements.

## Introduction

**Figure 1:**
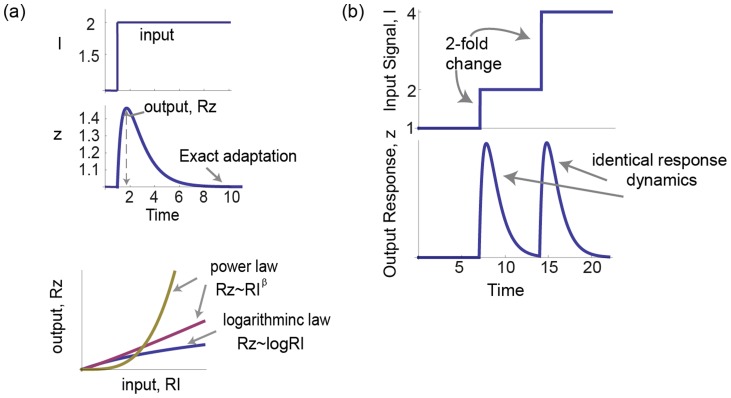
Input/output relationships of sensory systems can be described by a logarithmic law or a power law. a) In many sensory systems the dynamical response to a step increase in input signal, *I*, is a transient increase of output Z followed by adaptation to a lower steady state. The relative maximal response is 
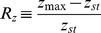
. Two laws are often found. The first is a logarithmic law, 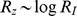. The second law is a power law, 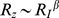 with different exponent 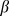 for each system. b) Fold change detection (FCD) describes a system whose response depends only on the relative change in input signal and not the absolute level. Therefore, for a step increase from 1 to 2 and then from 2 to 4 the system response curve is exactly the same.

Biological sensory systems have been quantitatively studied for over 150 years. In many sensory systems, the response to a step increase in signal rises, reaches a peak response, and then falls, adapting back to a baseline level, 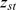 (Fig. 1a upper panel). Consider a step increase in input signal from 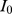 to 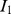, such that the relative change is 
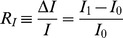
. There are two commonly observed forms for the input-output relationship in sensory systems: logarithmic and power law. In the logarithmic case, the relative peak response of the system 
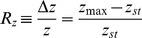
 is proportional not to the input level but to its logarithm 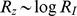. A logarithmic scale of z versus *I*, namely 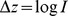, is often called the Weber-Fechner law [1], and is related but distinct from the present definition 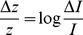. In the case of a power-law relationship, the maximal response is proportional to a power of the input 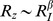 (Fig. 1a lower panel) [2]. In physiology this is known as the Stevens power law; the power law exponent 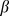 varies between sensory systems, and ranges between 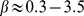 [2]. For example the human perception of brightness, apparent length and electrical shock display exponents 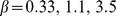 respectively.

Both logarithmic and power-law descriptions are empirical; when valid, they are typically found to be quite accurate over a range of a few decades of input signal. For example, both laws emerge in visual threshold estimation experiments [3]. In that study, the logarithmic law was found to describe the response to strong signals and the power-law to weak ones. However the mechanistic origins of these laws, and the mechanistic parameters that lead to one law or the other, are currently unclear. Theoretical studies have suggested that these laws can be derived from optimization criteria for information processing [4], [5], such as accounting for scale invariance of input signals [6]. Both laws can be found in models that describe sensory systems as excitable media [7]. Other studies attempt to relate these laws to properties of specific neuronal circuits [8], [9]. Here we seek a simple and general model of sensory systems which can clarify which mechanistic parameters might explain the origin of the two laws in sensory systems.

To address the input-output dependence of biological sensory systems, we use a recently proposed class of circuit models that show a property known as fold-change detection [10], [11]. Fold change detection (FCD) means that, for a wide range of input signals, the output depends only on the relative changes in input; identical relative changes in input result in identical output dynamics, including response amplitude and timing (Fig. 1b). Thus, a step in input from level 1 to level 2 yields exactly the same temporal output curve as a step from 2 to 4, because both steps show a 2-fold change. FCD has been shown to occur in bacterial chemotaxis, first theoretically [10], [11] and then by means of dynamical experiments [12], [13]. FCD is thought to also occur in human sensory systems including vision and hearing [11], as well as in cellular sensory pathways [14]–[17].

FCD can be implemented by commonly occurring gene regulation circuits, such as the network motif known as the incoherent feed-forward loop (I1-FFL) [10], as well as certain types of nonlinear integral feedback loops (NLIFL) [11]. Recently, the response of an FCD circuit to multiple simultaneous inputs was theoretically studied [18]. Mechanistically, FCD is based on an internal variable that stores information about the past signals, and normalizes the output signal accordingly. We find here, using analytical solutions, that simple fold-change detection circuits can show either logarithmic or power law behavior. The type of law, and the power-law exponent 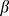, depend primarily on a single parameter: the steepness (effective Hill coefficient) of the effect of the internal variable on the output.

## Results

### Analytical solution for the dynamics of the I1-FFL circuit in its FCD regime

**Figure 2:**
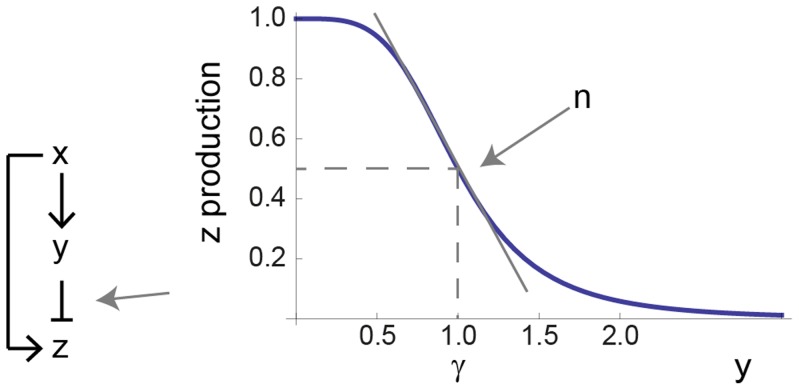
A model for the incoherent feed-forward loop includes three dimensionless parameters. In the incoherent type 1 feed-forward loop (I1-FFL) input X regulates an internal variable Y and both X and Y regulate Z. The repression of Z by Y is described by a Hill function with steepness *n* and halfway repression point 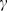.

We begin with a common gene regulation circuit [19] that can show FCD, the incoherent type 1 feed-forward loop (I1-FFL) [10]. In transcription networks, this circuit is made of an activator that regulates a gene and also the repressor of that gene. More generally, we can consider an input X that activates the output Z, and also activates an internal variable Y that represses Z (Fig. 2). We study a model (Eq. 1, 2) for the I1-FFL with AND logic (that is, where X and Y act multiplicatively to regulate Z), which includes ordinary differential equations for the dynamics of the internal variable Y and the output Z [20]–[22]. We use standard biochemical functions to describe this system [23].
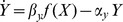
(1)

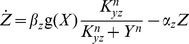
(2)The production rate of Y is governed by the input X according to a general input function 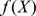 (in cases where X is a transcription factor, X denotes the active state). The maximal production rate of Y is 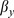. The repressor Y is removed (dilution+degradation) at rate 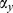 (Eq. 1). We assume here that saturating signal of Y is present, so that all of Y is in its active form. The product Z which is repressed by Y and activated by X is produced at a rate that is a function of both X and Y, denoted 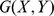. An experimental survey of *E. coli* input functions suggested that many are well described by separation of variables: the two-dimensional input function separates to a product of one dimensional functions, 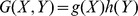 [24], where 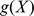 and 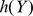 are Hill functions (for more explanation see the Methods section). We therefore use a general form for the X dependence, 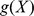, and multiply it by a repressive Hill function of Y (Eq. 2), with a maximal production rate 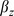. The removal rate of Z is 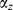. Here we consider step input functions in which X changes rapidly from one value to another. The values of 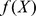 and 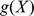 is determined by the step size in input.

For clarity, upper case letters relate to the elements in the circuit and lower case letters describe normalized model variables. The two-equation model (Eq. 1, 2) has 6 parameters. Dimensional analysis (fully described in Methods) reduces this to three dimensionless parameters (Eq. 3, 4).

The first parameter, 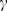, is the normalized halfway repression point of the output, defined by 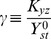, where 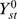 is the pre-step steady state level and 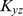 is the level of Y needed to half-way repress Z. The second parameter is the cooperativity or steepness of the input function, 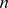. The final parameter is the ratio of decay rates of Z and Y, 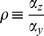. The normalized variables, 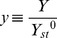 and 
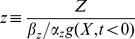
, are the new dimensionless variables in the model. Table 1 summarizes the parameters in the model for the I1-FFL.

**Table 1:** A parameter table for the I1-FFL model.

Parameter	Biological meaning	Definition
*β_y_*	Maximal production rate of Y	
*α_y_*	Removal rate of Y	
*β_z_*	Maximal production rate of Z	
*α_z_*	Removal rate of Z	
*K_yz_*	Halfway repression point of Z by Y	
*n*	Steepness of input function	
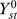	Pre-signal steady state of Y	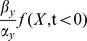
*γ*	Normalized halfway repression point of Z by Y (dimensionless)	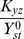
*ρ*	Removal rates ratio (dimensionless)	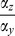

This model for the I1-FFL describes the response to a step increase in input, starting from fully adapted conditions. We consider a change between an input level of 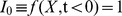, to a new level 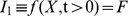. The step is thus characterized by the fold change *F* equal to the ratio between the initial and final input levels, 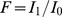.

In order for FCD to hold, the production rate of Z must be proportional to 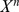 (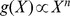), where the power law exponent 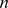 is the same as the Hill coefficient that describes the steepness of the input function. In this way, the internal variable, Y, can precisely normalize out the fold change in input (see Methods). The model thus reads:
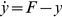
(3)

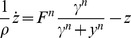
(4)The higher 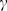, the more Y is needed to repress Z. The parameter 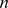 - the Hill coefficient of the input function - is important for this study, and determines the steepness of the regulation of the output Z by the internal variable Y (Fig. 2). The higher 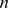 the more steep the repression of Z by Y. The limit 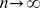 resembles step-like regulation. Biochemical systems often have Hill coefficients in the range 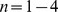 [23]. The ratio between the removal rates, 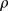, describes the relative time scale between Y and Z. For 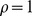, Y and Z have the same removal rates, and for 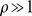, the output Z is much faster than Y.

**Figure 3:**
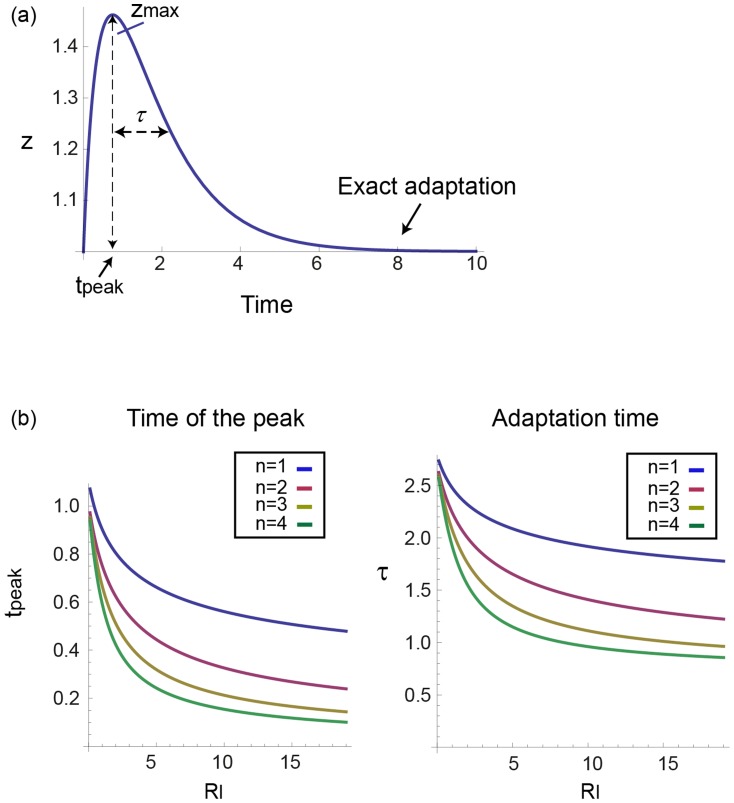
The I1-FFL shows FCD in the limit 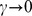. a) Response to a step increase in input from 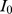 to 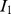, which can be described by 
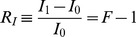
 where 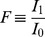. The output dynamics show three features of interest: the amplitude of the peak response 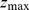 the timing of the peak, 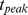 and the adaptation time 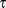. b) The time scales of the response, the timing of the peak 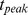 and the adaptation time 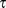, mildly decrease with the relative change in the input signal, 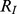. The steepness, *n*, does not have a dramatic affect on this decrease.

Goentoro et al. [15] showed, using a numerical parameter scan, that this circuit can perform FCD provided that threshold of the Z repression, 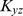, is small: that is 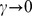. We therefore further analyze the limit of 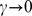, meaning strong repression of Z, where the equation for the product Z (Eq. 4) becomes:
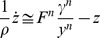
(5)In this limit, the system exhibits fold change detection since it obeys the sufficient conditions for FCD in Shoval et al (2010) (see Methods). We analytically solved the model (Eqs. 3, 5), in the limit of small 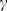, for all values of 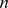, with initial conditions corresponding to steady state at the previous signal level, 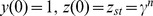 (in the limit 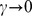). The solution (derived in Methods) is a decaying exponential multiplied by a term that contains a Beta function (Fig. 3a):
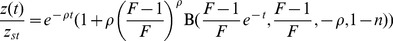
(6)where the Beta function is 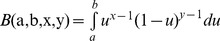. The dynamics of the output z shows a rise, reaches a peak 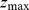, and then falls to the pre-signal steady state (Fig. 3a). At 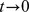 the solution is approximately linear with a slope that depends on *F*, 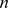 and 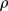:
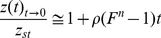
(7)At 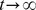 the solution decays exponentially:
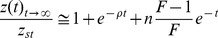
(8)As in all FCD systems, exact adaptation is found. The error of exact adaptation, 
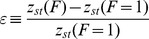
 goes as 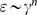 and vanishes at 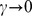.

We explored how three main dynamical features depend on the input fold change *F* and the dimensionless parameters 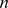 and 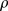. The first feature is the amplitude of the response, defined as the maximal point in the output z dynamics, 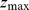. The second dynamical feature is the timing of the peak, 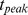. The third feature is the adaptation time, 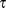 [25], [26] which we define as the time it takes z to reach halfway between 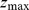 and its steady state (Fig. 3a). We denote 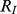 as the relative change in the input signal, 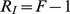 and 
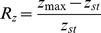
 as the relative maximal amplitude of the response. Since 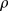 has only mild effects, we discuss it in the last section, and begin with 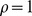, namely equal timescales for the two model variables.

### A power law relation emerges when the cooperativity *n* of the input function is larger than one; Logarithmic behavior occurs when *n* equals one

We tested the effects of cooperativity in the input function, 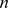, on the dynamics of the response. Cooperativity seems to have a weak effect on the timescales of the response: The adaptation time 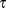 and the peak time 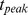 decrease mildly with the fold *F*. For 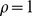, the analytical solution of the time of the peak 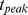 for all values of 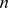 is: 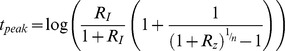 (see derivation in Methods). Substituting the corresponding relative response, 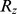, we receive a mildly decreasing function (Fig. 3b).

**Figure 4:**
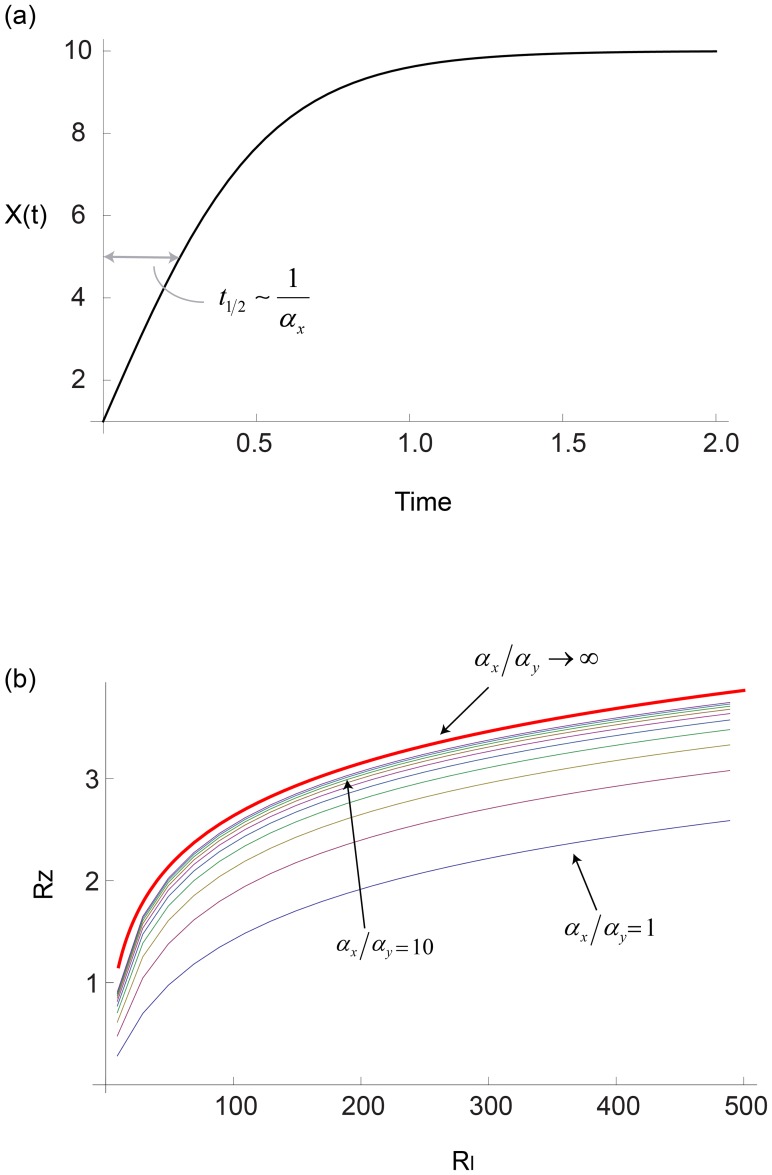
The response amplitude follows an approximately logarithmic law for *n* = 1 and a power law at *n*>1. a) The numerical solution of the amplitude of the response, 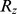, with *n* = 1, is shown (blue dots) as well as its analytical solution 
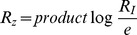
 (blue curve) in a log-linear plot. A fit to 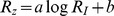 (red curve) captures the behavior better than a fit to 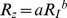 (green curve). b) For *n*>1 the numerical solution of the amplitude of the response, 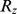, is shown (in dots) as function of 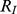 in a log-log plot. A fit to a power law behavior 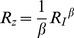 with only one parameter (solid lines) describes the numerical results better than a fit to a logarithmic behavior 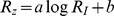 (dashed lines). At the limit of large 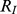, 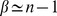.

In contrast to the mild effect of cooperativity on timescales, cooperativity has a dramatic effect on the response amplitude. The maximal amplitude of the output z relative to its basal level, 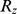, increases with the fold and behaves differently for each 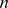. For low steepness, 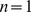, 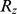 increases in an approximately logarithmic manner with 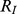 (for 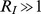), 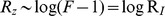 (normalized root-mean-square deviation, 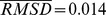 for fitting to 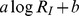 compared to 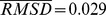 for fitting to 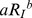
- see Methods). More precisely the analytical solution is 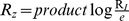 (see Methods) (Fig. 4a). The function 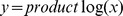 is defined as the solution to the equation 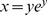. The productlog function is approximately linear at 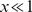, and approximately 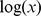 at 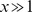.

For 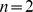, the peak response increases linearly 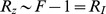. For 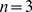, the increase is approximately quadratic, 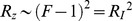 (Fig. 4b). We find that for any 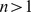, the increase is approximately a power law with exponent 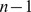 in the limit of large 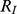: 
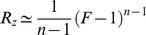
 (see Methods) (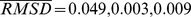 for fitting to 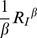 compared to 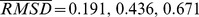 for fitting to 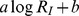 for 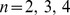 respectively). Note that the pre-factor in the power law is also predicted to depend simply on the Hill coefficient for 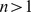, namely to be equal to 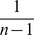 (for 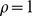). Indeed in fitting the numerical solution the best fit parameter is approximately 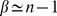: 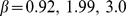 for 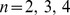 respectively. The dependence of output amplitude on input fold-change is thus a power law, similar to Stevens power law, except for 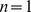 where the output dependence is logarithmic.

**Figure 5:**
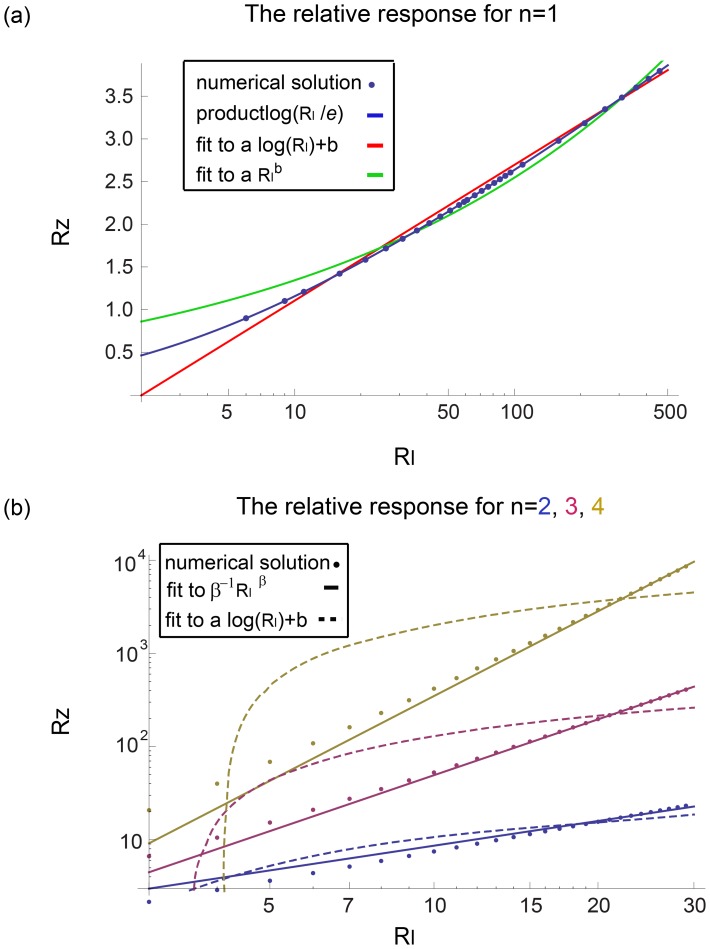
Rapidly changing input signal leads to responses similar to a step increase in signal; slowly changing input leads to weak response. a) Input signal with a tunable timescale, 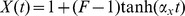 with 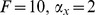. This signal goes form level 1 to level *F*, with a halfway time that goes as 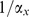. b) The relative maximal amplitude, 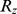, as a function of the relative change in the input signal 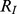, is plotted for various values of the input timescale 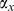. When the signal changes much faster than the timescale of the circuit, 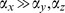, the response is similar to the analytical solution for an infinitely fast step in input (Full red curve). When the timescale is slow, 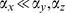, the response of the circuit is weak.

One point to consider regarding step input functions is that realistic inputs are not infinitely fast steps; however, a gradual change in input behaves almost exactly like an infinitely rapid step, as long as the timescale of the change in input is fast compared to the timescale of the Y and Z components. To demonstrate this, we computed the response to changes in input that have a timescale parameter 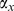 that can be tuned to go from very slow to very fast: 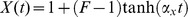 (Fig. 5a). When 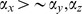, the behavior of the relative maximal amplitude of the response, 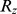, as a function of the relative change in the input signal, 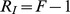, is very similar to the infinitely fast step solution (less than 5% difference for 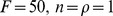 and 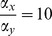, Fig. 5b). When the change in input is much slower than the typical timescales of the circuit, the response is very small, since the signal is perceived almost as a steady-state constant. For slow changes in input, the I1-FFL response can be shown to be approximately proportional to the logarithmic temporal derivative of the signal [27]–[30].

### A nonlinear integral feedback mechanism for FCD also shows a power law behavior

**Figure 6:**
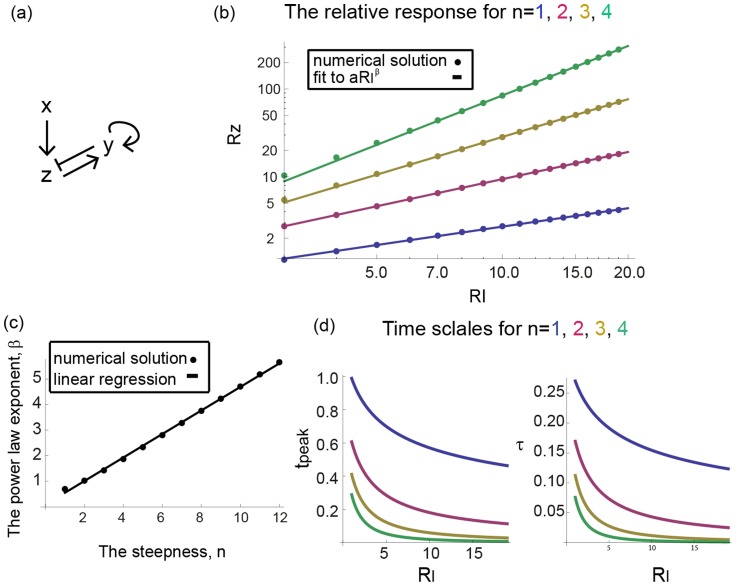
A different circuit showing FCD, the non-linear integral feedback loop (NLIFL), also exhibits a power law behavior. a) The NLIFL mechanism. b) The amplitude of the response is a power law of the relative change in input signal. c) The power-law exponent 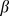 increases linearly with *n*. d) The time-scales decrease faster with the fold change of the signal, 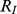, and with *n* than in the incoherent feed-forward loop case (Fig. 3b).

In addition to the I1-FFL mechanism, a non-linear integral feedback based mechanism (NLIFL) for FCD at small values of 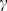 has been proposed by Shoval et al [11] (see Methods section) (Fig. 6a). This mechanism is found in models for bacterial chemotaxis [28]. The full model is described by:
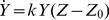
(9)

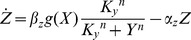
(10)Its dimensionless equations following dimensional analysis (fully described in Methods) are:
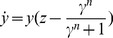
(11)

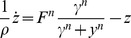
(12)Where the new variables are: 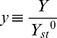, 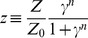 and the dimensionless parameters are defined as: 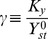 and 
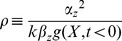
 (Methods). Table 2 summarizes the parameters in the model for the NLIFL.

**Table 2:** A parameter table for the NLIFL model.

Parameter	Biological meaning	Definition
*k*		
*Z*_0_	Steady state level of Z	
*β_z_*	Maximal production rate of Z	
*α_z_*	Removal rate of Z	
*K_y_*	Halfway repression point of Z by Y	
*n*	Steepness of input function	
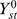	Pre-signal steady state of Y	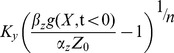
*γ*	Normalized halfway repression point of Z by Y (dimensionless)	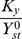
*ρ*	Timescale ratio (dimensionless)	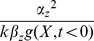

We solved the NLIFL model (Eqs. 11, 12) numerically for the limit 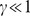 and find that the maximal response increases with the relative change in the signal in a power-law manner, 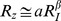 (Fig. 6b). The best-fit power law exponents increase with 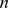, namely 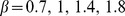 at 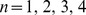 for 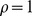. A 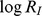 dependence does not fit the data at 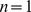 (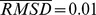 for fitting to 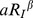 compared to 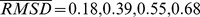 for fitting to 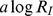 for 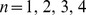 respectively). To a good approximation, the power law is linearly related to the steepness parameter 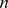, by 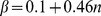 (Fig. 6c).

The time scales in this circuit seem to decrease faster with the fold *F* for 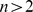 than in the I1-FFL case, 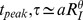 where 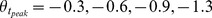 and 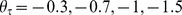 at 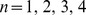 (Fig. 6d, all the fits of 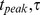 have 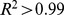).

Given the results so far, one can use the present approach to rule out certain mechanisms. If one observes a logarithmic dependence, one can draw at least two conclusions: (i) the NLIFL model addressed here can be rejected, (ii) if the I1-FFL model addressed here is at play, its steepness coefficient is 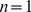.

If one observes a linear dependence of input on output, the I1-FFL and NLIFL mechanisms cannot be distinguished. The steepness can be inferred to be about 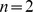 for both circuits.

### Logarithmic law in eukaryotic signaling FCD

**Figure 7:**
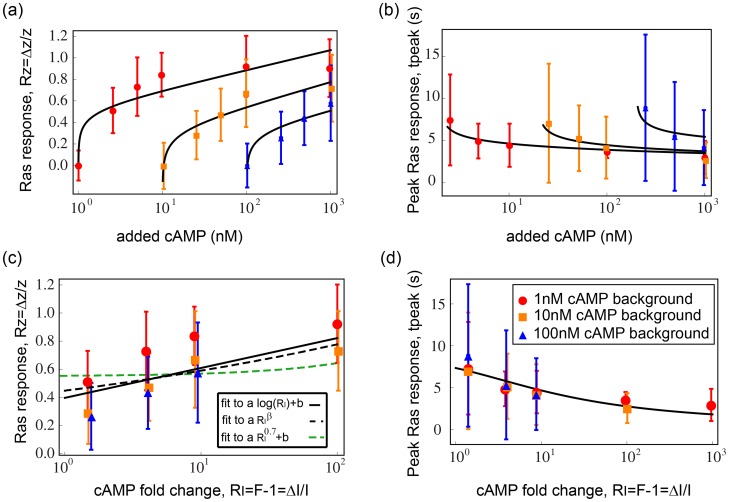
A mechanism of eukaryotic signaling FCD illustrates this theory. a) The response of Ras-GTP to different concentrations of added cAMP in Dictyostelium discoideum chemotaxis is re-plotted together with the timing of the peak (b). A logarithmic function describes the data well. The black lines are our fit to the data. c) The response of Ras-GTP is re-plotted as function of the different fold changes in cAMP concentrations. The solid line is a fit to 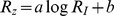, the black dashed line is a fit to 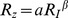 and the green dashed line is a fit to a power law with exponent 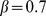. d) The corresponding solution for the timing of the peak for I1-FFL with *n* = 1 explains well the data.

We applied the present approach to data from Takeda et al [17] on *Dictyostelium discoideum* chemotaxis. In these experiments, the input is cAMP steps applied to cells within a micro-fluidic system, and the output is a fluorescent reporter for Ras-GTP kinetics. The output showed nearly perfect adaptation and FCD-like response to a wide range of input cAMP steps. We re-drew the peak amplitude (Fig. 7a) and the time of peak (Fig. 7b) as a function of the added cAMP concentrations and find that it is well described by the analytical solution of the maximal response and time of peak for an I1-FFL circuit with 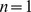. The peak amplitude (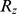) as a function of the relative input 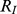 is well described by a logarithmic relationship (mean-square weighted deviation, 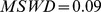 for fitting the data to 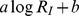 considering the error-bars – see Methods). Fitting it to a power law 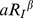 results in a small exponent 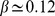 (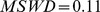) (Fig. 7c). Such a small power law exponent can only be obtained with a negative cooperativity in the NLIFL model considered here. Such negative cooperativity is rare in biological systems [31], [32]. If we consider only positive cooperativity (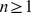), as found in most biological systems, the NLIFL model considered here provides a poor fit to the data (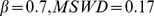) (Fig. 7c).

Thus, the present analysis is most consistent with an I1-FFL mechanism considered here with 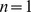. The same is found when plotting the observed time-to-peak (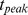) versus the analytical solution of the I1-FFL model (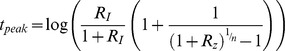) with 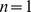 (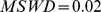 for fitting to 
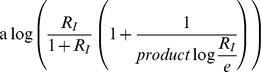
) (Fig. 7d). This agrees with the numerical model fitting performed by Takeda et al, who conclude that an I1-FFL mechanism is likely to be at play (they used 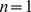 in their I1-FFL model, which is based on degradation of component Z by Y, rather than inhibition of production of Z by Y as in the present model).

In this analysis we assumed that the experimentally measured fluorescent reporter is in linear relation to the biological sensory output, Ras activity. If this relation turns out to be nonlinear, the conclusions of this analysis must be accordingly modified.

### Effect of timescale separation between the variables

**Figure 8:**
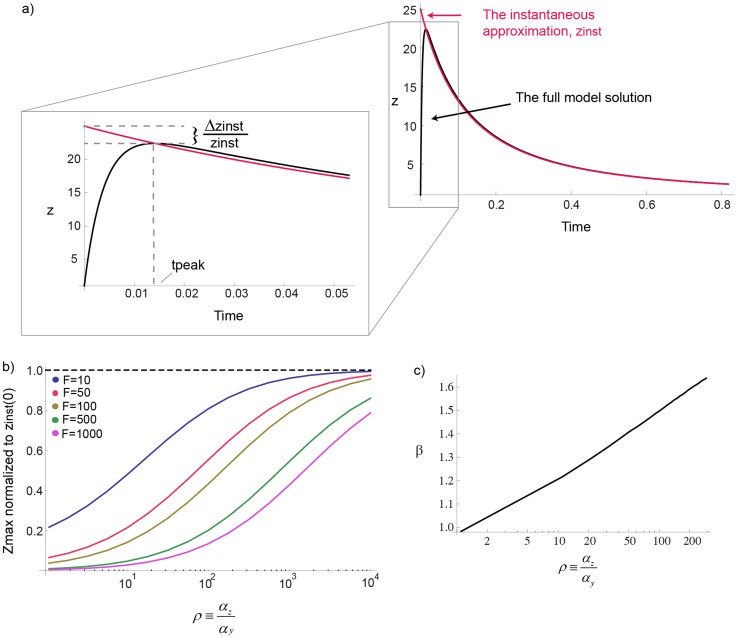
The instantaneous approximation does not capture the correct amplitude behavior. a) The power law for *n* = 1 increases mildly with 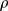 to a value between 1 and 2. b) The instantaneous approximation (in red) and the full model solution (in black) are plotted as function of time for 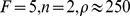. c) The maximal response 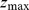 normalized to 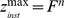 is plotted for different folds and for *n* = 1. The error between the maxima of the instantaneous approximation and the full model increases with the fold *F*.

In the eukaryotic chemotaxis system, the two model variables Y and Z have similar timescales (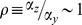). We also studied the effect of different timescales (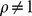), and find qualitatively similar results. A logarithmic dependence of amplitude on *F* is found when 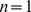, and a power law when 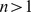. The power law 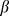 increases weakly with 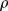 (Fig. 8a). In the limit of very fast Z (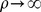), the solution approaches an instantaneous approximation (obtained by setting 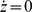) in which the power law is 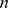 instead of 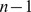 (Fig. 8b). There is a cross over from the Stevens power law 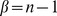 when 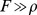, to the instantaneous model power law 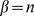 when 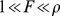 (Fig. 8c). An analytical solution that exemplifies this crossover can be obtained at 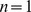, where 
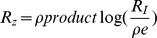
 (Methods). Because of the limit behavior of the *productlog* function mentioned above, at small fold values 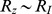, and at large values 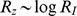. In summary, the instantaneous approximation, commonly used in biological modeling, must be done with care in the case of FCD systems.

## Discussion

This study explored how two common biophysical laws, logarithmic and power-law, can stem from mechanistic models of sensing. We consider two of the best studied fold-change detection mechanisms, and find that a single model parameter controls which law is found: the steepness 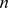 of the effect of the internal variable on the output. We solved the dynamics analytically for the I1-FFL mechanism, finding that logarithmic-like input-output relations occurs when 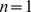, and power-law occurs when 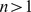, with power law 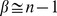, and prefactor 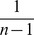 at 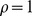. The nonlinear integral feedback loop (NLIFL) mechanism - a second class of mechanisms to achieve FCD - can only produce a power law. Thus, if one observes logarithmic behavior, one can rule out the specific NLIFL mechanism considered here. This appears to be the case in experimental data on eukaryotic chemotaxis [17], in which good agreement is found to the present results in the I1-FFL mechanism with 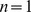 in both peak response and timing.

This theory gives a prediction about the internal mechanism for sensory systems based on the observed laws that connect input and output signals. Thus, by measuring the system response to different folds in the input signal one may infer the cooperativity of the input function and potentially rule out certain classes of mechanism. For example, if a linear dependence of amplitude on fold change is observed (power law with exponent 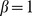), one can infer that the steepness coefficient is about 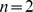 for both the specific I1-FFL and NLIFL circuits considered here, with slight modification if the timescales of variables are unequal. Such a linear detection of fold changes may occur in drosophila development of the wing imaginal disk [33]–[35].

The problem of finding the FCD response amplitude shows a feature of technical interest for modeling biological circuits. In many modeling studies, a quasi-steady-state approximation, also called an instantaneous approximation, is used when a separation of timescales exists between processes. In this approximation, one replaces the differential equation for the fast variables by an algebraic equation, by setting the temporal derivative of the fast variable to zero. This approximation results in simpler formulae, and is often very accurate, for example in estimating Michaelis-Menten enzyme steady states [36]. However, as noted by Segel et al [36], this approximation is invalid to describe transients on the fast time scale. In the present study, we are interested in the maximal amplitude of the FCD circuits. In some input regimes, namely 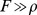, the instantaneous approximation predicts an incorrect power law. To obtain accurate estimates, the full set of equations must be solved without setting derivatives to zero.

It would be interesting to use the present approach to analyze experiments on other FCD systems, and to gain mechanistic understanding of sensory computations.

## Methods

### The two dimensional input function can be considered as a product of one dimensional input functions

Consider a general partition function for an input function with an activator and a repressor: 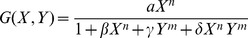. The regime in which FCD applies is that of strong repression, 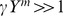 and non-saturated activation 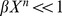 [10]. In this limit, 
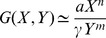
, and is thus well approximated by a product.

More generally, G(X,Y) is a product of two functions whenever binding is independent, 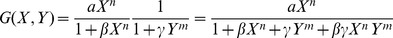, which occurs when the relation 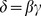 holds. The biological meaning of the relation is that X and Y bind the Z promoter independently so that the probability of X to bind the promoter and the probability of Y to unbind equals the multiplication of the probabilities:



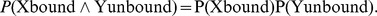
In the NLIFL case, one can show from the MWC model chemotaxis by Yu Berg et al [28] that in the FCD regime it is simply a power law.

### Dimensional analysis of the full model for I1-FFL and NLIFL

We performed dimensional analysis of the full model of the I1-FFL (Eq. 1, 2) by rescaling as many variables as possible. The rescaled variables:
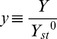
(M1)

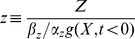
Where 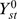 is the pre-signal steady state of Y, derived by taking 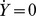: 
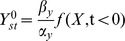
, and 
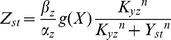
 is the steady state of Z derived by taking 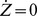. Substituting these rescaled variables we receive:
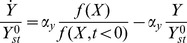
(M2)

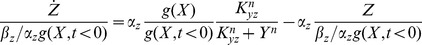
Since we assume that 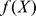 is determined by the step size in input, we can consider merely the fold change *F* in input, 
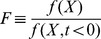
. For FCD to hold we consider 
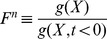
. Defining the rescaled repression threshold 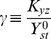 we receive in the new rescaled variables (lower case letters y and z):
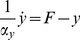
(M3)

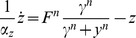
Rescaling the time to 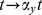 and defining 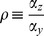 yields to Eq. 3, 4 in the main text.

We also performed dimensional analysis of the full model of the NLIFL (Eqs. 9, 10) by rescaling as many variables as possible. The rescaled variables:
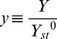
(M4)

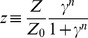
Where 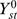 is the pre-signal steady state of Y, derived by taking 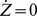 and assuming 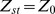 : 
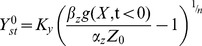
, and 
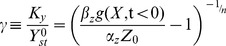
. Substituting these rescaled variables we receive:
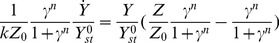
(M5)

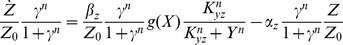
After algebraic manipulation and in the new rescaled variables (lower case letters y and z):
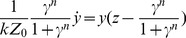
(M6)

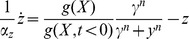
We consider here also 
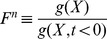
.

Rescaling the time to 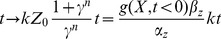 and defining 
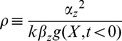
 yields to Eqs. 11, 12 in the main text.

### Proof that FCD holds in the model for I1-FFL and NLIFL

Given a set of ordinary differential equations with internal variable y, input *F* and output z:
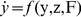
(M7)

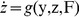
(M8)According to Shoval et. al. (2010), FCD holds if the system is stable, shows exact adaptation and g and f satisfy the following homogeneity conditions for any 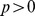:
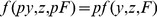
(M9)

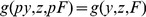
(M10)In the model for I1-FFL (Eq. 3, 4) at the limit of strong repression 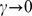:
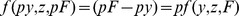


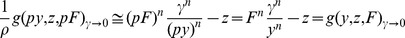
Exact adaptation also holds at 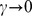, 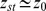. This holds also for the NLIFL (Eqs. 9, 10).

### Analytical solution for the I1-FFL

The solution for y is an exponent:
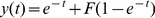
(M11)The general solution for the ODE 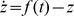 with the initial condition 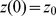 is:
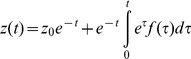
(M12)For our model Eq. M12 reads:
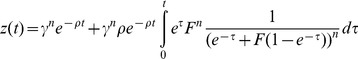
(M13)By changing the variable in the integral in Eq. M13: 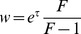 we get:
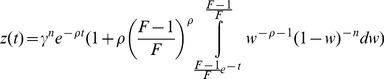
(M14)Which is by definition the solution in Eq. 6.

### Analytical solution for the time of peak for the I1-FFL

At the time of peak 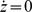, therefore from Eq. 5 in the main text we get:
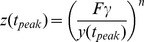
(M15)From our definition of the relative response 
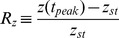
 we have:
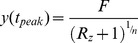
(M16)Substituting the solution of y (Eq. M11) and by algebraic manipulation we receive the analytical solution for 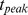:
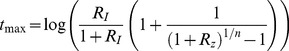
(M17)


### Analytical solution for the maximal response

The analytical results were derived by taking the derivative of the solution for 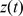 (Eq. 6 in the main text) and substituting time of the peak (Eq. M17), 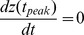. This provides an equation for the amplitude of the maximal response, 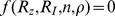, yielding an intractable equation:
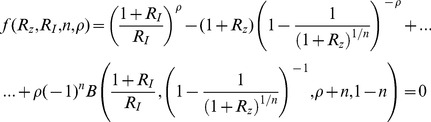
(M18)Where we used the identity: 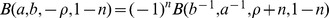. This identity can be easily proven by using the change of variable, 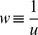, in the integral of the Beta function.

For 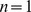 Eq. M18 becomes:
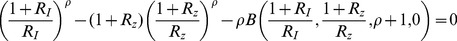
(M19)Using the Series function of Mathematica to expand Eq. M19 in the limit of large 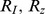 and keeping high orders in 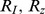 yields:
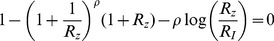
(M20)Using 
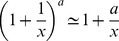
 in the limit of large x we receive:
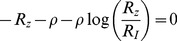
(M21)Taking the exponent of this Eq. M21 yields:
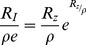
(M22)The solution for Eq. M22 is by definition the productlog function: 
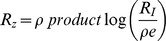
.

For 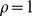 Eq. M18 becomes:
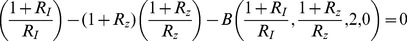
(M23)Since 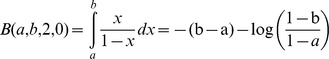, Eq. M23 yields:
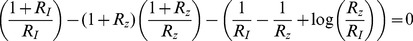
(M24)By algebraic manipulation Eq. M24 becomes 
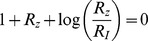
. Taking the exponent of this equation yields:
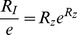
(M25)The solution for Eq. M25 is by definition the productlog function: 
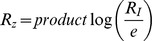
.

For 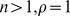 we define 
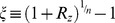
, substituting this new variable into Eq. M18 we have:
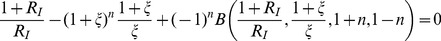
(M26)Using the Series function of Mathematica for large 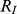 and 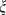 yields:
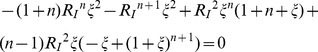
(M27)Keeping the highest order in 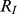 and 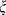 we receive: 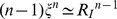. Recall that 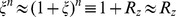 for large 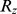 and 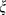, and therefore 
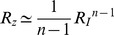
.

### The instantaneous approximation does not capture the correct amplitude behavior

For the instantaneous approximation to be true at large 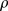, the error, 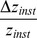 (Fig. 8a), between the maximal amplitude in the instantaneous approximation and the full model should vanish at 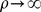.
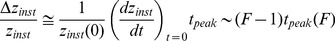
(M28)Where 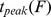 decrease with *F* slower than 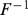, therefore 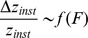 with *f*(*F*) a monotonic increasing function of *F*. This proves that even at large 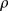, the error increases with *F* (Fig. 8b) and can be very large.

### Fits and numerical simulations

All the numeric simulations and fits were made in Mathematica 9.0.

The root-mean-square deviation (RMSD) [37] calculated for comparing the goodness of fit between the two models is defined as: 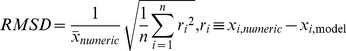.

The data points from Takeda et al were extracted by using the ‘ginput’ function of MATLAB. The fits for the data were made using the NonlinearModelFit function considering the error-bars, 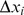, as weights, 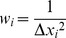.

The goodness of fit was tested using the mean-square weighted deviation (MSWD) [37] which sums the residuals (r) - sum of squares of errors with weights of 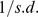: 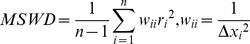.

### Note on biophysical law terminology

We define logarithmic response as 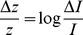. In contrast, traditional definition of the Weber-Fechner law (also called the Fechner law) in biophysics is (e.g. ref. [3]) as 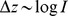. Thus the present definition concerns relative change in input and output, whereas the Weber-Fechner law concerns absolute input and output. Note also that the Weber-Fechner law is distinct from Weber's law, on the just noticeable difference in sensory systems, whose relation to FCD was discussed in Ref [11].
